# Onderdiagnostiek bij kanker door de COVID-19-crisis naar diagnose, leeftijd en provincie: Een cruciale rol voor eerstelijnszorgverleners

**DOI:** 10.1007/s12508-020-00289-1

**Published:** 2020-12-11

**Authors:** Carin A. Uyl-de Groot, Melinda S. Schuurman, Peter C. Huijgens, Jaike Praagman

**Affiliations:** 1 Erasmus School of Health Policy & Management, institute for Medical Technology Assessment, Erasmus University Rotterdam, Rotterdam, Nederland; 2 Integraal Kankercentrum Nederland, Utrecht, Nederland

**Keywords:** Symptomen, Prognose, Huisartsen, Vertrouwen, Drempel, Symptoms, Prognosis, General practitioners, Faith, Hurdle

## Abstract

De COVID-19-crisis en de intelligente lockdown hebben ertoe geleid dat het Nederlandse zorgsysteem maandenlang voor niet-COVID-patiënten op slot kwam te zitten. Patiënten durfden of konden niet naar hun huisarts, huisartsen waren terughoudend met doorverwijzingen naar het ziekenhuis en de zorg- en diagnostische processen werden vertraagd of aangepast. Dit gold ook voor kankerpatiënten. Hoe ernstig deze onderdiagnostiek is voor de prognose van de kankerpatiënt, hangt vooral af van kenmerken van de betreffende soorten kanker. In dit onderzoek hebben we de onderdiagnostiek in kaart gebracht met behulp van gegevens uit de Nederlandse Kankerregistratie en het Pathologisch-Anatomisch Landelijk Geautomatiseerd Archief. Vanaf de week waarin de eerste COVID-19-patiënt in Nederland werd gediagnosticeerd, is een daling van 20–40% in het aantal kankerdiagnoses te zien. De daling geldt voor vrijwel alle tumorsoorten, ook voor kankers waarbij het niet tijdig stellen van de diagnose levensbedreigend is, zoals bij patiënten met kanker in de luchtwegen (gemiddeld 23%) en het hoofd-halsgebied (gemiddeld 36%), en bij hematologie (gemiddeld 26%). Er moet meer aandacht komen voor de gesignaleerde onderdiagnostiek en de rol van de eerstelijnszorgverleners, zoals huisartsen en tandartsen. Daarbij is het zeer belangrijk dat patiënten altijd het vertrouwen houden dat ze bij klachten hun zorgverlener kunnen raadplegen.

## Abstract (en)

The COVID-19 crisis and the intelligent lockdown have led to a situation in which the Dutch health care system was locked for months for non-COVID-19 patients. Patients did not dare to go to their general practitioner, general practitioners were reticent about hospital referrals, and diagnostic and treatment trajectories were delayed or adapted. This also concerned cancer patients. The severity of the underdiagnosis for these patients mainly depends on their prognosis. In this study, we evaluated the magnitude of underdiagnosis using data from the Netherlands Cancer Registry and the Dutch registry of histo- and cytopathology (PALGA). From the week of the first COVID-19 diagnosis in the Netherlands, a decrease of 20–40% in the number of cancer diagnosis was noticed. This decrease was observed in nearly all cancer types, including cancers such as lung cancer (average 23%), head-neck cancer (average 36%) and hematology (average 26%), for which a missed or delayed diagnosis can be life threatening. Therefore, we want more attention for the observed underdiagnosis and the role of the primary care givers, including the general practitioner and dentist. Furthermore, it is of crucial importance that patients with symptoms or complaints feel no hesitation to visit their caregivers.

## Inleiding

De COVID-19-crisis en de intelligente lockdown, die verdere verspreiding in Nederland moet voorkomen, hebben ertoe geleid dat het Nederlandse zorgsysteem maandenlang voor niet-COVID-patiënten op slot kwam te zitten. De prioriteit in de zorg kwam te liggen op het creëren van voldoende capaciteit van de intensive care (IC), de zwakste schakel in het zorgsysteem tijdens de COVID-19-pandemie.

Al begin maart 2020 liet het Integraal Kankercentrum Nederland (IKNL) op basis van data uit de Nederlandse Kankerregistratie (NKR) en het Pathologisch-Anatomisch Landelijk Geautomatiseerd Archief (PALGA) zien dat het aantal kankerdiagnoses aanzienlijk was gedaald [1, 2]. De oorzaak moet liggen in uitgestelde diagnostiek. Een dergelijke *delay* kan worden veroorzaakt door de (potentiële) patiënt, de zorgverlener en/of het zorgsysteem. Het stellen van vragen over de redenen van uitstel is voor de toekomst extreem belangrijk, om zo uitstel te voorkomen. In het geval van COVID-19 is er een aantal factoren aan te wijzen (zie voor beschrijving ook Dinmohamed et al. [1]). Zo kunnen individuen met niet-specifieke symptomen barrières ondervinden en een huisarts niet consulteren vanwege morele bezwaren. Ze willen de tijd van de huisarts niet belasten met niet-COVID-19-gerelateerde symptomen, denken dat er niet voldoende zorgcapaciteit over is voor niet-COVID-19-gerelateerde klachten en/of zijn bang om COVID-19 op te lopen in de spreekkamer. Daarnaast bleken huisartsen terughoudend en lieten ze patiënten minder snel naar het spreekuur komen en vervingen ze normale spreekuren gedeeltelijk door teleconsulten. Deze consulten zijn geschikt voor niet-acute zaken. De huisarts kan immers geen lichamelijk onderzoek doen en zal een patiënt niet op zijn spreekuur vragen als de symptomen niet duidelijk naar een potentiële kankerdiagnose wijzen. Ook waren ziekenhuizen terughoudender met consulten en ontstond er ‘verstopping’ bij het aanvragen en beoordelen van diagnostische tests doordat veel capaciteit werd ingenomen door de zorg voor COVID-19-patiënten. Tot slot zijn ook de nationale screeningsprogramma’s voor borst-, dikkedarm- en cervixkanker met ingang van 16 maart een tijd stopgezet om het gezondheidszorgsysteem te ontlasten.

Tijdens de eerste intelligente lockdown daalde het aantal huisartsconsulten met 70% en het aantal verwijzingen naar het ziekenhuis met 75%, en halveerde het aantal nieuwe kankerdiagnoses [3–5].

Hoe ernstig deze onderdiagnostiek is voor de prognose van de kankerpatiënt, hangt vooral af van kenmerken van de betreffende kankers. Zo maakt het uit in welk stadium de kanker zich bevindt, wat de groeisnelheid en agressiviteit van de kanker is en wat de behandelmogelijkheden zijn voor de betreffende patiënt. Voor kanker met een relatief gunstige prognose, zoals borst- en huidkanker, is een tijdelijk delay mogelijk minder ‘erg’ dan voor ziekten waarvan snelle, tijdige diagnostiek van levensbelang kan zijn. We denken hierbij aan aandoeningen als long-, keelholte-, slokdarm-, alvleesklier-, eierstok- en maagkanker, en een aantal hematologische kankers [6].

Het is belangrijk om eerst inzicht te krijgen in de mate van onderdiagnostiek in zijn totaal, om vervolgens de mate van onderdiagnostiek voor diverse subgroepen in kaart te brengen. Inzichten in onderdiagnostiek voor de diverse tumorsoorten en regio’s kunnen bijvoorbeeld voorspellen waar er later een piek in het aantal diagnoses te verwachten is. Daarnaast kunnen we mogelijk ook lessen trekken voor de toekomst op basis van de genoemde factoren en andere factoren, zoals leeftijd.

## Methode

We hebben patiënten geïncludeerd uit de Nederlandse Kankerregistratie (NKR) van wie op basis van diagnoses uit PALGA bekend is dat ze in de eerste 23 weken van 2020 (tot en met 6 juni) gediagnosticeerd zijn met een invasieve vorm van kanker (data-extractie vond plaats op zondag 21 juni 2020). Voor vergelijking is gekeken naar de diagnoses uit dezelfde periode in 2019 om de mogelijke invloed van andere factoren, zoals jaargetijde en vakantieperiodes zichtbaar te maken. Er is een identieke data-extractie uit de NKR gemaakt op zondag 23 juni 2019. Dit was noodzakelijk omdat de diagnoses die momenteel van 2020 bekend zijn, grotendeels bestaan uit voorlopige registraties en in de loop van de tijd nog kunnen veranderen nadat aanvullend onderzoek is verricht. Dit laatste heeft vooral invloed op de stadiëring van de kanker.

De analyses zijn beschrijvend van aard. We hebben gekeken naar de relatieve verandering in kankerdiagnoses tijdens de COVID-19-periode. Hiervoor is het aantal kankerdiagnoses per week vergeleken met het gemiddelde aantal kankerdiagnoses in week 2 tot en met 5 van 2020 en uitgedrukt als percentage van dit gemiddelde. Naast het totale aantal diagnoses is er een uitsplitsing gemaakt naar type kanker, leeftijdscategorie (<65, 65–74, 75–84, ≥85 jaar) en provincie.

Voor de gestratificeerde analyses is het totale aantal diagnoses van meerdere weken genomen (maximaal vier opeenvolgende weken), omdat de aantallen per week te weinig inzicht kunnen geven in de trends van diverse subgroepen. De aantallen zijn hier ook weer vergeleken met het aantal diagnoses in week 2 tot en met 5. Voor de uitsplitsing naar tumorsoort is gekozen voor een indeling op algemene hoofdgroep, met uitzondering van kanker in de mondholte/orofarynx en hematologische maligniteiten. Voor andere kankersoorten werden de aantallen per week bij verdere opsplitsing te klein om trends te kunnen weerspiegelen.

## Resultaten

**Figure 1:**
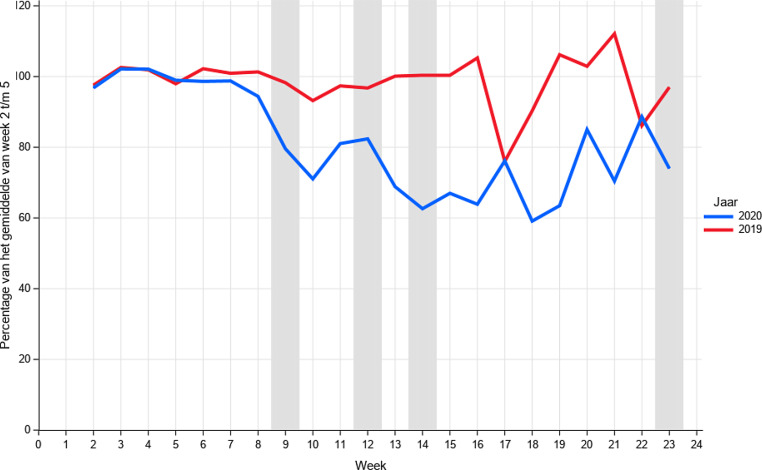

In fig. 1 is voor week 2 (6 januari) tot en met week 23 (6 juni) het relatieve percentage nieuwe kankerdiagnoses weergegeven. Vanaf week 9, de week waarin de eerste COVID-19-patiënt in Nederland werd gediagnosticeerd, is een duidelijke daling in het aantal kankerdiagnoses te zien. In vergelijking met de eerste weken van 2020 blijkt dat er tijdens de piek van de pandemie 20–40% minder kankerdiagnoses zijn gesteld. Een start van het herstel lijkt te zien vanaf week 20, maar het aantal diagnoses blijft in verhouding laag. Een dergelijke daling is niet te zien in 2019, op week 17, 18 en 22 na, een periode met veel vakantie- en feestdagen. In totaal gaat het om 5.000 gemiste kankerdiagnoses.

**Figure 2:**
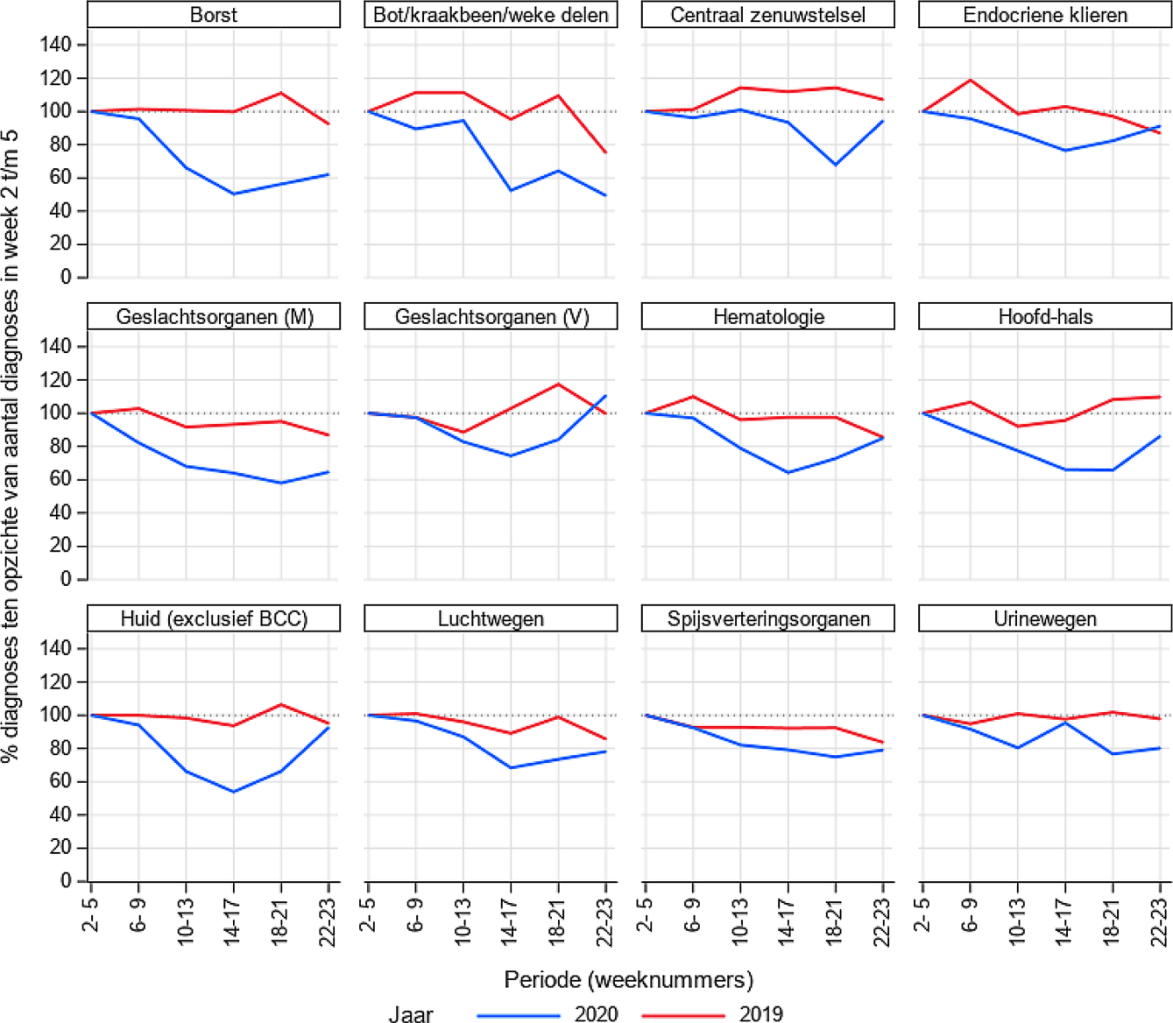

De daling is voor vrijwel alle tumorsoorten te zien (fig. 2), met de duidelijkste dalingen voor huid- en borstkanker. Voor de meeste lokalisaties neemt het aantal diagnoses in de loop van de periode weer toe, al lijkt dit minder het geval voor borstkanker, bot/kraakbeen- en wekedelenkanker, en kanker van de mannelijke geslachtsorganen. Kankers met een hogere mate van risico op levensbedreigende situaties door uitgestelde diagnose, zoals bij luchtwegen, hoofd-hals en hematologie, laten ook een aanzienlijke daling zien.

**Figure 3:**
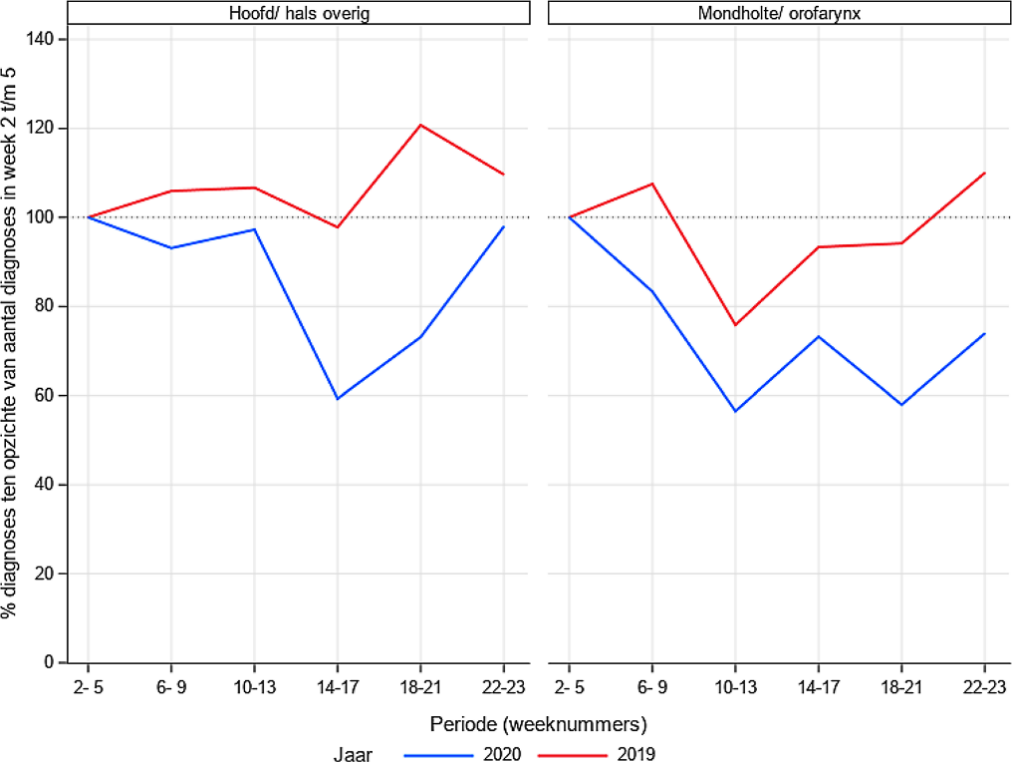

In fig. 3 en 4zijn de resultaten voor hoofd-halstumoren en hematologie opgesplitst. De hoogrisicogroepen, dat wil zeggen patiënten bij wie de prognose slecht is als niet zo snel mogelijk een therapie wordt ingezet, betreffen kanker van de mondholte/orofarynx en agressief lymfoom/acute leukemie. Ook hier is een daling in aantal gediagnosticeerde risicopatiënten waar te nemen, van gemiddeld 36% voor mondholte/orofarynx, 15% voor agressief lymfoom en 26% voor acute leukemie.

**Figure 4:**
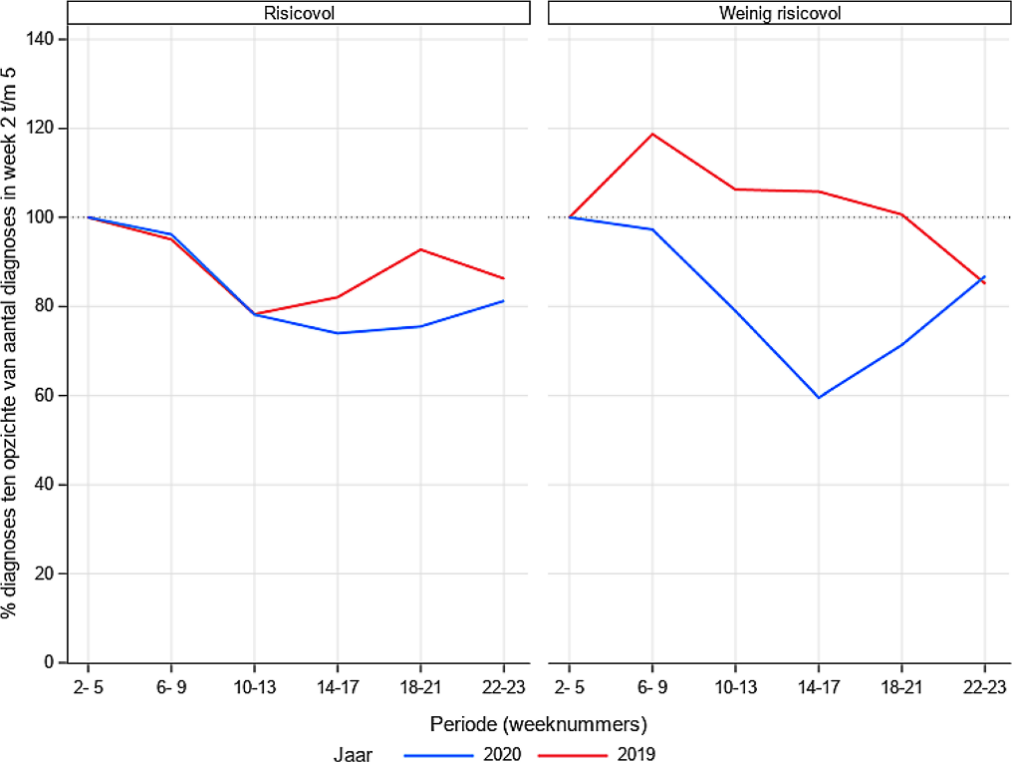

**Figure 5:**
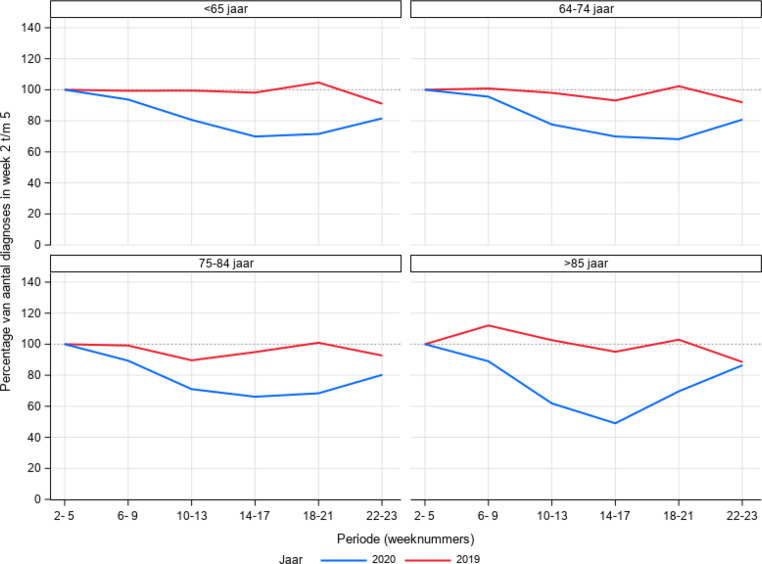

De gesignaleerde daling geldt voor alle leeftijdscategorieën (fig. 5), maar is het grootst in de groep boven de 85 jaar.

**Figure 6:**
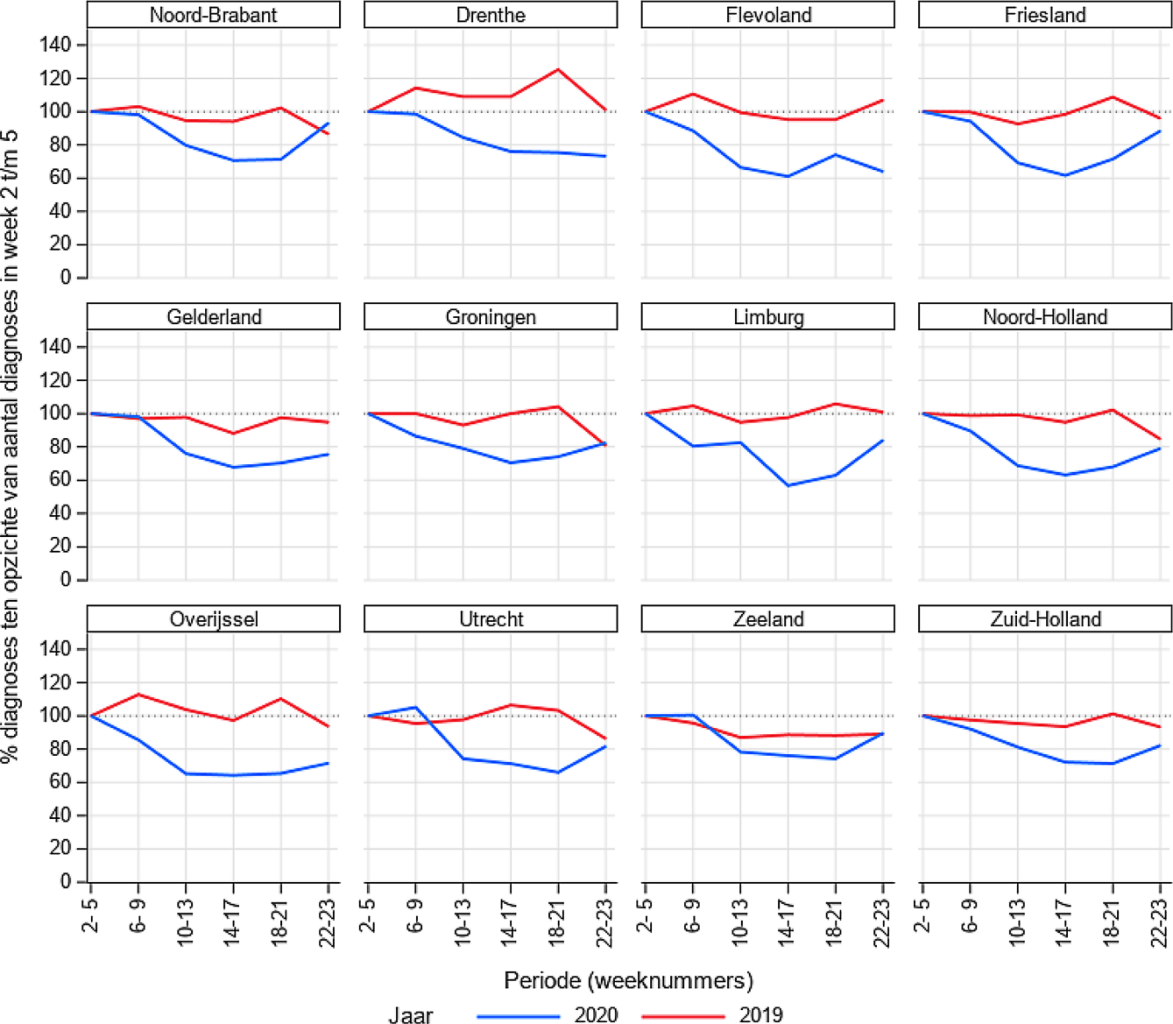

De daling is in alle provincies te zien (fig. 6), inclusief de noordelijke provincies, waar een relatief laag aantal COVID-19-besmettingen heeft plaatsgevonden [7].

## Beschouwing

Dit onderzoek beschrijft de relatief hoge mate van onderdiagnostiek in Nederland tijdens de eerste paar maanden van de COVID-19-pandemie. Dit is te zien voor alle kankersoorten, alle leeftijden en in alle provincies. Voor enkele groepen, zoals kankers van de luchtwegen, het hoofd-halsgebied en hematologie, is het missen van diagnoses van direct levensbelang.

Deze daling zal grotendeels toe te schrijven zijn aan de COVID-19-pandemie, maar er zijn wel week tot week fluctuaties zichtbaar. In de eerste weken van het jaar (onze referentieperiode) liggen de aantallen doorgaans wat hoger vanwege een inhaalslag na de kerstvakantie. Ook spelen feestdagen een rol in de fluctuaties. Voor kleinere tumorsoorten is het ook lastiger om conclusies te trekken. Daarom hebben we de trends met 2019 vergeleken en meerdere weken samengenomen.

De bevolkingsonderzoeken voor borst-, baarmoederhals- en darmkanker zijn stopgezet in week 12, maar er zit een aantal weken tussen het bevolkingsonderzoek en de pathologische bevestiging van een tumor [2]. Het effect van het staken van de screening zou vanaf week 14 zichtbaar kunnen zijn. De dalingen in het aantal diagnoses, vooral bij borstkanker, vonden echter al eerder plaats.

In week 15 hebben diverse partijen, waaronder IKNL, gezamenlijk een oproep gedaan om zich met klachten eerder bij de huisarts te melden. Dit heeft bij geen van de onderzochte groepen een duidelijk effect gehad [8, 9]. Een belangrijke vraag is hoe we deze mate van onderdiagnostiek dan wel zo snel mogelijk kunnen inhalen en hoe we deze nu in een nieuwe golf of in een volgende pandemie van respiratoire virussen kunnen minimaliseren. Hiervoor moeten de redenen van de genomen maatregelen en de gevolgen ervan goed in kaart worden gebracht.

Diverse factoren kunnen mogelijk bijdragen aan het voorkomen van het wegblijven van mogelijke patiënten bij de eerstelijnszorg. Ten eerste is het van belang dat patiënten voldoende kennis van mogelijke klachten en symptomen hebben en dit benadrukken als ze hulp zoeken, bijvoorbeeld door de huisarts te bellen. Klachten variëren uiteraard per tumorsoort en kunnen ook bij andere ziekten voorkomen. Voor diverse tumorsoorten is het ook lastig om de diagnose te stellen, omdat er (nog) geen klachten of symptomen zijn. Bij andere soorten zijn er klachten die om alertheid vragen. De volgende klachten kunnen wel duiden op een maligniteit: bloed in slijm, ontlasting of urine, (moeder)vlekken die nieuw zijn of veranderen, een verdikking of knobbeltje op de huid of in het lichaam, gewichtsverlies of moeheid zonder reden [10]. KWF Kankerbestrijding adviseert om met deze klachten naar de huisarts te gaan, wanneer ze langer dan vier weken duren [10]. Het is dan ook belangrijk dat hier nu meer aandacht aan wordt besteed, bijvoorbeeld via voorlichtingscampagnes, zodat patiënten sneller hulp zoeken. Eerstelijnszorgverleners, zoals huisartsen, tandartsen en mondhygiënisten, spelen een belangrijke rol bij de diagnostiek en juist daarom is aandacht voor hun rol erg belangrijk. Patiënten dienen altijd het vertrouwen te houden dat ze hun hulpverlener bij klachten (veilig) kunnen opzoeken. Dit geldt zeker voor patiënten die klachten hebben die kunnen duiden op longkanker, mondholtekanker en bepaalde hematologische maligniteiten. Volgens clinici betekent groei van de kanker bij deze tumorsoorten een lagere kans op genezing, meer morbiditeit (mondholte) of een grotere kans op snelle dood (acute leukemie).

Op het moment dat we dit schrijven vindt er opnieuw afschaling van de reguliere zorg plaats vanwege een tweede COVID-19-piek. Op- en afschaling van de zorg gebeurt aan de hand van een lijst met gestelde diagnoses waarbij tijdige diagnostiek dus niet als criterium is opgenomen [5, 9]. De lijst gaat immers uit van diagnoses en de diagnose is dus al gesteld. Uit ons onderzoek blijkt dat er veel onderdiagnostiek is. Daarom pleiten we voor meer aandacht voor diagnostiek, voor een inhaalslag ervan en voor en het vermijden van nieuwe onderdiagnostiek.

Ook in andere landen heeft de COVID-19-pandemie geleid tot een delay in kankerdiagnoses. In de Verenigde Staten is het aantal kankerdiagnoses tot eind april 2020 met 65% verminderd [11, 12]. Een deel hiervan is veroorzaakt door uitstel van screening.

In welke mate de extra delay door de COVID-19-pandemie impact heeft op de uitkomsten voor de patiënt is nog niet duidelijk. Dit zal ook afhangen van andere factoren, waaronder het type kanker, het stadium waarin de kanker zich bevindt, de groeisnelheid en agressiviteit van de tumor, hoe goed het type kanker te behandelen is, maar ook of en hoeveel later de patiënt uiteindelijk de diagnose heeft gekregen. Dergelijke vragen, en de vraag welke impact de COVID-19-pandemie op de behandeling van kankerpatiënten heeft gehad, zullen in vervolgonderzoeken worden beantwoord.

In het Verenigd Koninkrijk zijn reeds modelmatige berekeningen gedaan naar de impact van de COVID-19-epidemie op de delay in kankerdiagnoses en de veranderingen in de zorgverlening, berekend vanaf het punt van de diagnosestelling. In deze berekeningen zijn ook het aanpassen, uitstellen of weglaten van behandelingen meegenomen [13]. De auteurs hebben de impact van diverse scenario’s op de overleving van patiënten met respectievelijk borst-, colorectaal-, long- en slokdarmkanker doorgerekend. In het meest conservatieve scenario wordt bij borstkanker geschat dat er over vijf jaar 7,9% meer patiënten zullen overlijden. Voor colorectaal-, long- en slokdarmkanker bedroegen deze percentages respectievelijk 15,3%, 4,8% en 5,8%. In totaal schat men dat er voor deze vier tumorsoorten als gevolg van de eerste COVID-19-crisis na 5 jaar minimaal 3.300 patiënten extra overleden zullen zijn [13].

Nu we in de tweede COVID-19-golf zitten, zou er meer aandacht aan de diagnostiek van andere ziekten, zoals kanker, besteed moeten worden. In de zomer was het aantal kankerdiagnoses ongeveer gelijk aan het aantal van 2019 en in september is er een inhaalslag geweest [14]. Op dit moment zien we echter weer een terugval in het aantal huisartsconsulten [15]. Het is dan ook cruciaal dat zorgverleners weten dat ze ook de andere zorg op een veilige manier kunnen verlenen en daarmee levens kunnen redden of verlengen. Hierbij moet wel aangetekend worden dat we in sommige ziekenhuizen weer een ondercapaciteit zien. De crux blijf echter dat patiënten eerst bij de huisarts of andere eerstelijnszorgverleners moeten komen, voordat ze überhaupt doorgestuurd kunnen worden. De eerstelijnszorgverleners, zoals huisartsen, tandartsen en mondhygiënisten, vormen bij het oplossen van de gesignaleerde onderdiagnostiek dan ook een belangrijke schakel.

## Besluit

In dit onderzoek laten we zien dat het aantal kankerdiagnoses tijdens de eerste lockdown is achtergebleven bij wat we verwachtten. Wat de exacte gevolgen hiervan zijn moet nader onderzoek uitwijzen. Het is belangrijk dat de drempel om eerstelijnszorg te zoeken voor patiënten wordt weggenomen en dat de reguliere zorg zo goed mogelijk doorgang moet vinden.
